# Burden of Alcoholic Liver Disease: Bhutan Scenario

**DOI:** 10.5005/jp-journals-10018-1267

**Published:** 2018-05-01

**Authors:** Pelden Wangchuk

**Affiliations:** Eastern Regional Referral Hospital, Monggar, Bhutan

**Keywords:** Alcohol, Bhutan, Disease burden, Liver diseases.

## Abstract

Alcoholic liver disease (ALD) is one of the major public health problems in Bhutan. The incidence of alcohol liver diseases (per 10,000 populations) in the year 2016 was 46.

The burden of mortality associated with it is alarming, going by the size of the population of the country. It is one of the all-time top five killer diseases in the country. In 2016, the mortality attributable to alcohol liver disease was 184 corresponding to 97% of deaths due to reported liver diseases. The ALD is responsible for 15% of all deaths on an average in the last 3 years.

**How to cite this article:** Wangchuk P. Burden of Alcoholic Liver Disease: Bhutan Scenario. Euroasian J Hepato-Gastroenterol 2018;8(1):81-82.

## INTRODUCTION

Bhutan is a tiny Himalayan country located between the two Asian giants of India in the south and China in the north. Most of the health indicators have improved over the years since the country opened itself to the outside world in 1961, when it initiated its first 5-year plan. Its average life expectancy at birth has increased from 32 years in 1960 to 70 years at present.^1^

Bhutan is traditionally an alcohol-consuming country. Mean household alcohol consumption was 31.72 and 22.3 L in 2003 and 2007 respectively, with per capita adult pure alcohol consumption of 8.47 L.^2^ This is much higher than the global per capita of 6.2 L. Some attributable factors are availability of a wide variety of cheap alcoholic beverages, cultural acceptability, and extreme cold weather.

Like any country, alcohol-related problems are not different for its population. It ranges from addiction, dependency to major health problems like cirrhosis and death. It also causes social issues like family disharmony, poverty, crime, and accidents apart from economic loss.^2^

Alcoholic liver disease is one of the major public health problems in Bhutan. The incidence of alcohol liver diseases (per 10,000 populations) in the year 2016 was 46 (Table 1).^3^

**Table 1:** **Table 1:** Incidence of ALD in Bhutan, 2012 to 2016^4^

		*Year*									
*Indicator*		*2012*		*2013*		*2014*		*2015*		*2016*	
ALD incidence (per 10,000 population)		29		36		42		41		46	

**Table 2:** **Table 2:** Top five causes of mortality in Bhutan, 2014 to 2016

		*Year*					
*Disease*		*2016*		*2015*		*2014*	
ALD		184		153		156	
Cardiovascular disease		255		245		152	
Septicemia		140		136		69	
Cancer		126		106		47	
Pneumonia		76		57		57	

**Graph 1:**
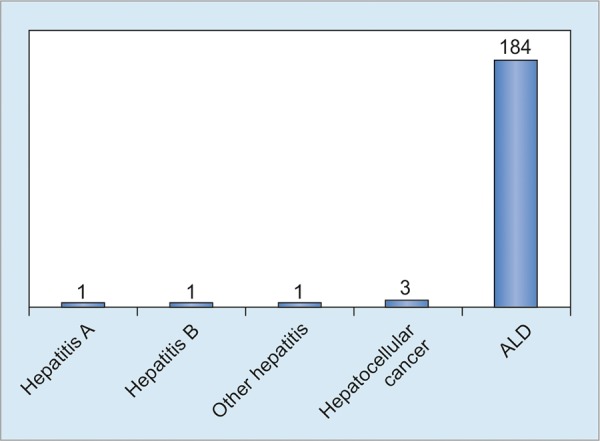
Mortality due to liver diseases, 2016

The burden of mortality associated with it is alarming, going by the size of the population of the country. It is one of the all-time top five killer diseases in the country (Table 2).^4^ In 2016, the mortality attributable to alcohol liver disease was 184 corresponding to 97% of deaths due to reported liver diseases. The ALD is responsible for 15% of all deaths on an average in last 3 years (Graph 1).

The ALD also drains the economy. The expenditure incurred was about Nu. 48.94 million during 2007. This amount was spent on outpatient, inpatient, and referral abroad (Table 3).

Looking at the pattern of alcohol use, it is almost an inseparable part of our culture and tradition. Table 4 shows different alcohol use categories and their significance.

Further, alcohol offered to a guest has a corresponding name according to his activities of the day. The alcoholic beverage offered soon after getting up in the morning is called zheng chang. Likewise, pheb chang for the beverage offered on arrival, zhuk chang for beverage offered soon after the guest sits down, toh chang for beverage offered just before a meal, and zim chang for beverage just before the bedtime. This practice is mostly prevalent in the rural eastern part of the country. This shows that alcohol use is deeply entwined with the culture and tradition of the country.

Some of the measures the Royal government has undertaken in the last decades to mitigate the problems of ALD are: Banning of household alcohol production, increased taxation on factory-produced alcohol, specified legal age of consumption of alcohol from 18 to 21 years of age, and identified and monitored dry day a week on Tuesdays, among others. In recent years, the Ministry of Health also has conducted rounds of advocacy on the ill effects of alcohol, targeting population in the rural as well as urban areas. It is an ongoing activity for most of the health assistants tied to their individual workplan.

Although the government has initiated various measures to control alcohol use by its population, much more needs to be done to prevent alcohol-related problems in the future.

**Table 3:** **Table 3:** Proportion of deaths due to ALD, 2014 to 2016

*Year*		*Deaths due to ALD*		*Deaths due to all other causes*		*% death due to ALD*	
2014		176		806		17.9	
2015		158		1066		12.9	
2016		184		1151		13.7	

**Table 4:** **Table 4:** Traditional alcohol use category^2^

Serkem chang		Drink offerings to local deities	
Tor chang		Drinks furnished while making ritual cakes	
Deutsi chang		Spiritual offertory drinks	
Yang chang		Brewed for the god of wealth	
Ngo chang		Drink offerings for the sake of the dead	
Tsan chang		Drink offerings to local deity	
Tshe chang		Drinks brewed for long life rituals	
Tshog chang		Communal tradition of offering drinks to visitors (popular in east)	
Duen chang		Drinks to welcome guests (pastoral societies)	
